# Umbilical cord hemangioma and pseudocyst with favorable fetal outcome

**DOI:** 10.1002/ccr3.7656

**Published:** 2023-07-04

**Authors:** Sikolia Wanyonyi, Felix Nyagaka, Patricia Okiro, Lilian Ogutu, Alice Nyaichowa, Felix Oindi, Evan Sequeira

**Affiliations:** ^1^ Department of Obstetrics and Gynecology Aga Khan University Nairobi Kenya; ^2^ Department of Pathology Aga Khan University Nairobi Kenya

**Keywords:** fetal outcome, hemangioma, pseudocyst, tumor, umbilical cord

## Abstract

**Key Clinical Message:**

There is a high association between umbilical cord hemangiomas or cysts with fetal mortality. However, favorable outcome is possible with proper prenatal monitoring and care

**Abstract:**

Umbilical cord hemangiomas are rare neoplasms of vascular origin, commonly found in the free section of the umbilical cord proximal to placental insertion. They are associated with an increased risk of fetal mortality. We present a rare co‐occurrence of an umbilical cord hemangioma and a pseudocyst managed conservatively, with favorable fetal outcome despite the interval increase in size, decreased caliber of the umbilical arteries, and fetal chest compression.

## Graphical abstract

An umbilical pseudocyst and hemangiona compressing the arteries but with positive end diastolic flow.

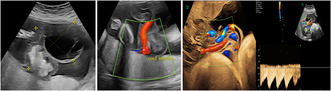

## 1 CASE PRESENTATION

A 33‐year‐old gravida 2 woman, with well‐controlled early onset gestation hypertension, presented at 20 weeks for an anomaly scan. Her antenatal profile and aneuploidy screening were unremarkable. Her first pregnancy was complicated with pre‐eclampsia at term, but had a spontaneous vaginal delivery, following induction of labor with good outcome.

**FIGURE 1:**
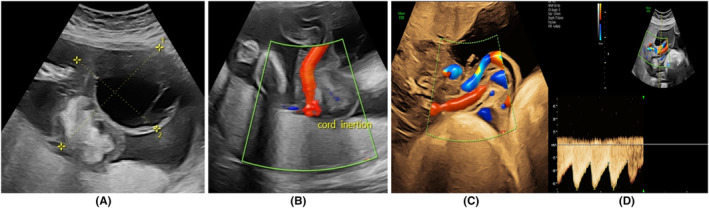
(A, B) Antenatal ultrasound image showing cystic and solid areas. (B) Cord insertion. (c) Note the difference in the calibers of the umbilical arteries arrows. (D) Positive end diastolic flow.

On the anomaly scan, a large complex multi‐cystic mass with solid components was noted proximal to the cord insertion (Figure 1A,B). The lesion measured 10.91 cm × 8.22 cm. Both umbilical arteries had thickened tunica with positive end diastolic flow (EDF). However, there was discordance in velocity of blood flow of the umbilical arteries (14 and 49 cm/s) (Figure 1C,D). The umbilical cord was markedly edematous with increased Wharton's jelly. The rest of the fetal anatomy was normal.

**FIGURE 2:**
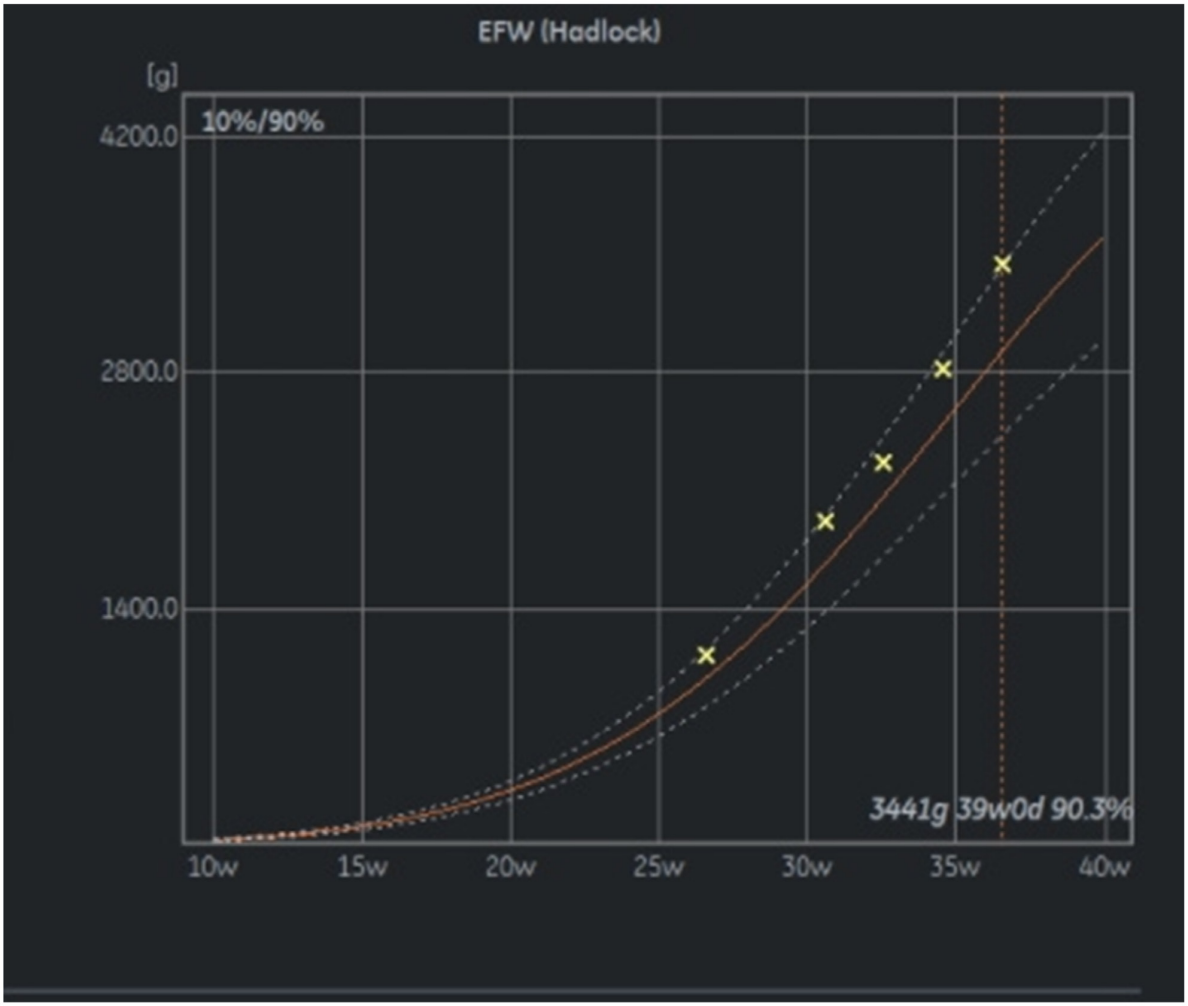
Fetal growth velocity from 28 weeks.

Follow‐up ultrasound done every 2 weeks showed an interval increase in size of the solid components. Despite the variance in caliber and velocity of the umbilical arteries, the positive EDF in the vessels persisted till delivery. From 32 weeks onward, the mass appeared to compress the chest, but there were no signs of obstruction, and the lung volumes remained normal for gestational age. The abdominal circumference and estimated fetal weight were above the 90th centile and the amniotic fluid volume was normal (Figure 2). An elective cesarean delivery was planned at 38 weeks due to uncertainty on the ability of the fetus to tolerate labor and possibility of intrapartum death. The decision was arbitrary and was made after discussion with the patient on potential outcomes based on previous case reports.^1^, ^2^, ^3^, ^4^

**FIGURE 3:**
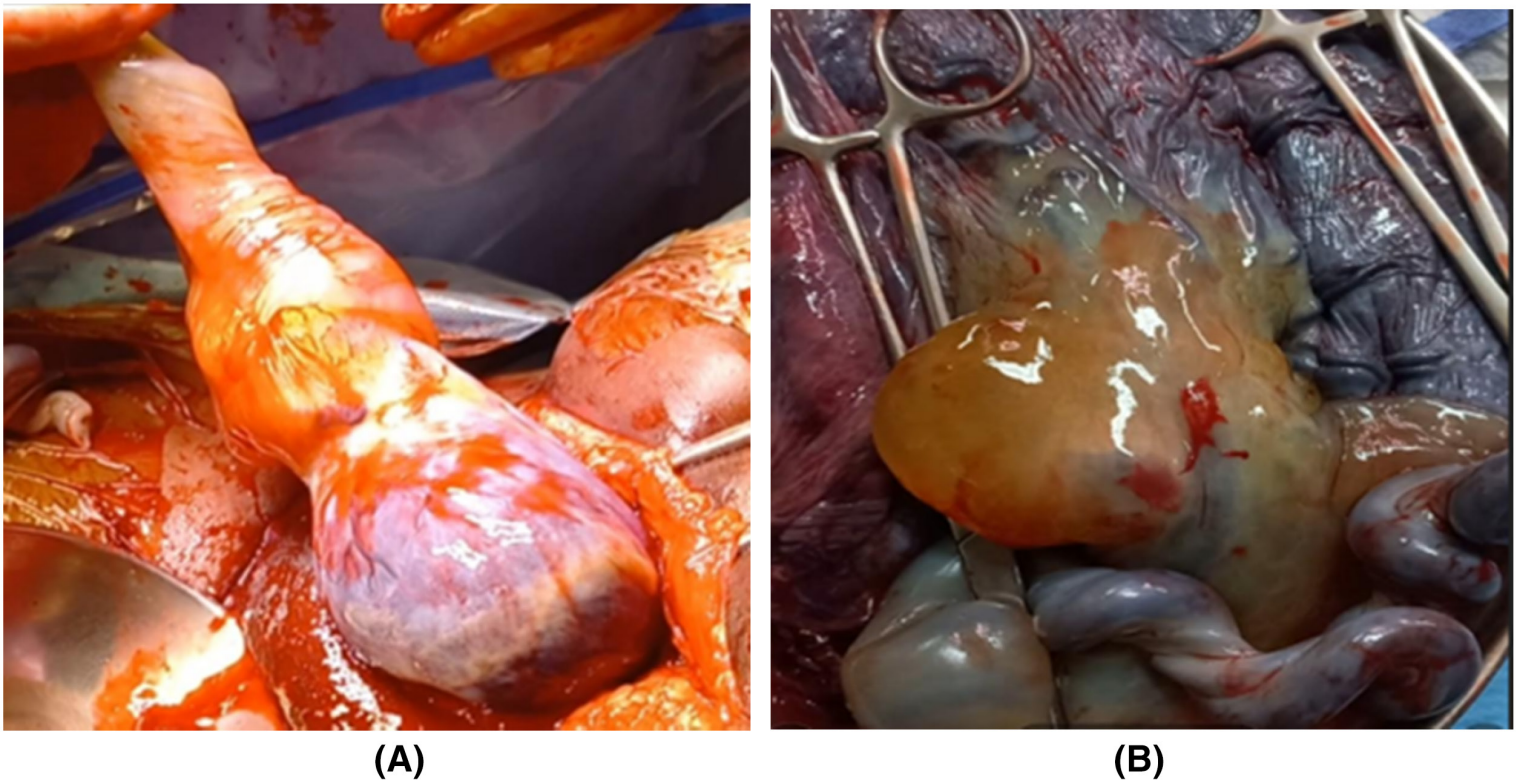
(A) Solid mass near placental end with ruptured cyst. (B) Fluid‐filled cyst and hypercoiled cord.

On admission, a computed cardiotocogram (CTG) performed was normal. A live male infant was subsequently delivered with a birthweight of 3480 g and an Apgar score of 10 at 5 min. The umbilical cord cyst ruptured during delivery. The placenta spontaneously separated. The umbilical cord mass was noted (Figure 3A,B). The placenta was taken for histopathological examination and immunohistochemistry.

**FIGURE 4:**
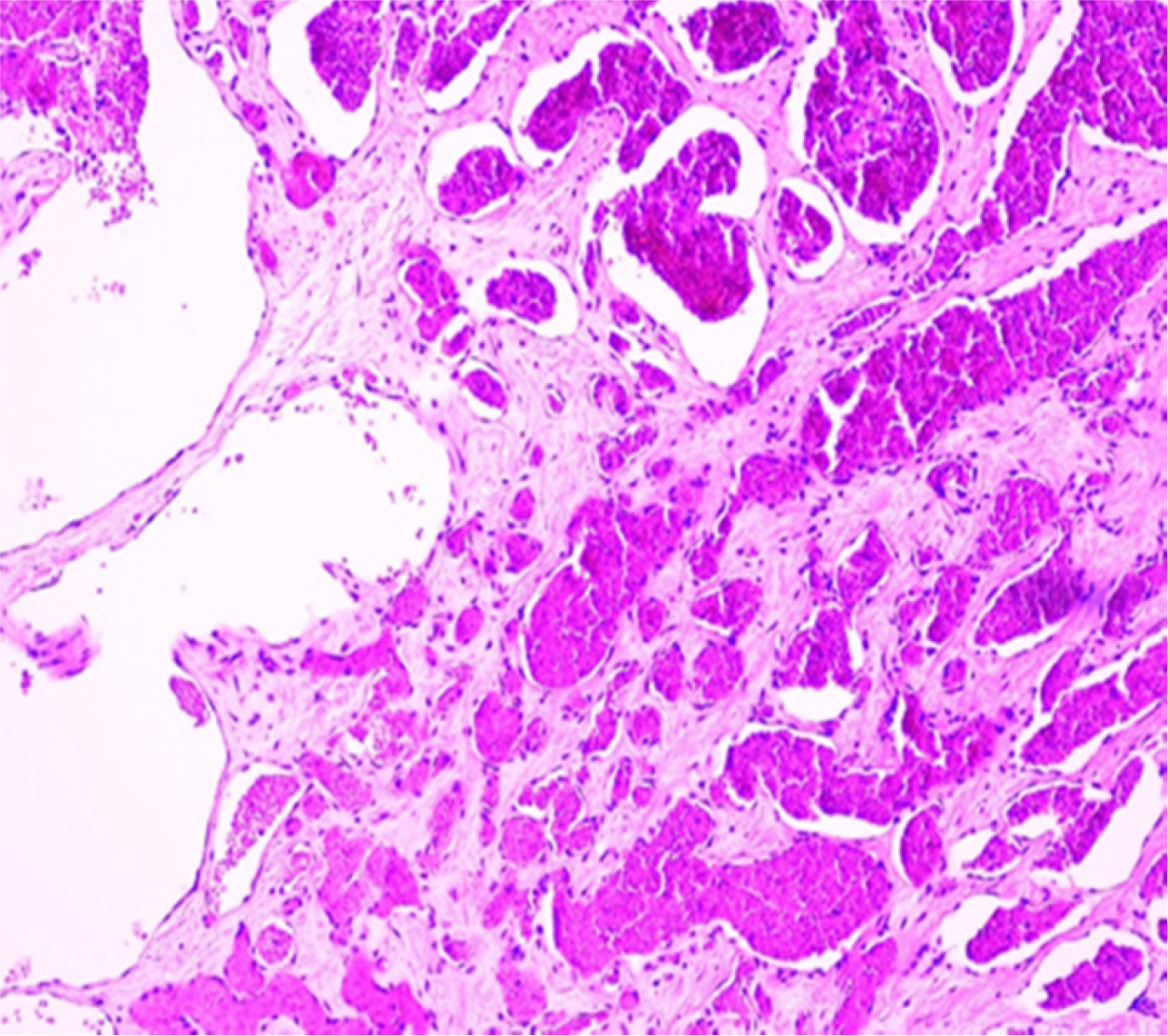
H&E‐stained section depicting the hemangioma.

The gross formalin‐fixed specimen showed a translucent and hyper‐coiled cord with prominent vascular pattern and a multinodular mass at the insertion site measuring 7 × 7 × 4 cm and a collapsed cyst measuring 15 × 10 cm (Figure 4A). Upon dissection, a variegated lobular lesion with peripheral hemorrhage measuring 3.8 × 2.2 cm was seen (Figure 4B). The diameter of the cord at the lesion site was 5.7 cm. The lesion was limited to the umbilical cord.

Histology confirmed an umbilical hemangioma with a pseudocyst, mild acute funisitis, intervillous hematomas, delayed villous maturation and avascular fibrotic villi (Figure 4C). The findings were collaborated on CD34 immunohistochemistry staining (Figure 4D).

## 2 DISCUSSION

Umbilical cord hemangiomas are rare benign endothelial‐derived neoplasms composed of blood vessels. Typically, hemangiomas are found in skin and soft tissues, but their presence in other organs has been demonstrated.^1^ Umbilical hemangiomas tend to have a predilection for the placental end of the cord and are likely to arise from one or more of the major umbilical vessels.^2^, ^3^, ^4^, ^5^, ^6^ Umbilical cysts may present as true cysts, resulting from damage to the amniotic surface of the cord by presence of the hemangioma. The epithelial‐lined cysts are embryological remnants of either the allantois or omphalomesenteric duct. On the other hand, pseudocysts arise from degeneration of Wharton's jelly surrounding the cord vessels.^2^ Differential diagnoses include cord teratomas, aneurysms, omphalomesenteric duct cysts, allantoic cysts, hematomas and varicose veins, abdominal wall defects, and metastatic neuroblastoma.^2^ Although no clear causal relationship is established, an association between cord hemangiomas and increased risk of fetal mortality has been exhibited. So far, no successful in utero intervention has been described.^3^

Prenatal ultrasound can reliably differentiate hemangioma and pseudocyst from other umbilical cord tumors.^7^ The presence of positive EDF of the umbilical artery Doppler with normal pulsatility index and progressive fetal growth provides buoyancy to prolong the pregnancy.^4^, ^8^

Even though rare, umbilical cord neoplasms are predominantly hemangiomas and less commonly teratomas.^1^, ^2^ Hemangiomas arise from endothelial cells, especially of the umbilical arteries, and have an undefined pathophysiology.^9^ In a unique occurrence, Thayer et al. recently documented an umbilical cord hemangioma occurring synchronously with maternal COVID‐19 infection with adverse fetal outcome.^1^

Hemangiomas may present as fusiform swellings of the cord comprising of an angiomatous nodule, surrounded by edema of the Wharton's jelly with myxoid cystic degeneration. They are clearly demarcated from surrounding stroma. These nodules vary in dimensions, ranging from 0.2 to 7 cm in largest dimension, and if associated with edema may measure up to 18 cm.^1^, ^2^, ^3^, ^9^ A hypercoiled umbilical cord was noted with a multinodular mass at the insertion site. A collapsed cyst 15 cm × 10 cm, a residual nodule 7 cm × 7cm × 4 cm with an edematous cord were also seen and this is in keeping with dimensions of other hemangiomas reported. Extensive myxoid degeneration of Wharton's jelly was also confirmed in our case.

A strong association exists between cord hemangioma and intrauterine fetal demise (IUFD). Possible reasons include vascular compression, intravascular thrombosis, intravascular tumor proliferation, and hemorrhage from ruptured vessels. The risk of IUFD is highest if compression of the umbilical veins is present.^3^, ^4^, ^10^, ^11^ Umbilical cord pseudocysts have also been linked to aneuplodies.^12^ In our case the fetus was euploid and had a good outcome despite reduced velocity in one of the umbilical arteries and interval increase in size of the lesion. The flow in the umbilical veins remained unaffected.

Management of fetuses with umbilical cord hemangiomas depends on gestational age and early delivery may reduce the risk of IUFD, but the decision is weighed against possible complications of prematurity.^3^, ^4^, ^11^ In our case, the patient had serial ultrasound scans done every 2 weeks to monitor the velocity of the umbilical arteries. The decision to delivery at 38 weeks was based on the compressive effect observed, maternal hypertension, and uncertainty on the outcome. We managed our case on an outpatient basis for logistic reasons; however, it must be borne in mind that there are high chances of sudden fetal demise and as such consideration should be given to closer monitoring of such patients in the hospital.

## 3 CONCLUSION

The outcome of cord hemangiomas is not well understood, and neither is their pathophysiology elaborated. However, despite its high association with fetal mortality, a favorable outcome is possible with close monitoring of the umbilical artery Doppler flows and timely delivery, the size of the lesion notwithstanding.

## AUTHOR CONTRIBUTIONS

**Sikolia Wanyonyi:** Conceptualization; supervision; validation; writing – original draft; writing – review and editing. **Felix Nyagaka:** Conceptualization; formal analysis; writing – original draft; writing – review and editing. **Patricia Okiro:** Formal analysis; resources. **Lilian Ogutu:** Formal analysis; resources. **Alice Nyaichowa:** Conceptualization; formal analysis. **Felix Oindi:** Resources; writing – review and editing. **Evan Sequeira:** Conceptualization; supervision; writing – review and editing.

## CONFLICT OF INTEREST STATEMENT

The authors have no conflict of interest to declare.

## CONSENT

Written informed consent was obtained from the patient to publish this report in accordance with the journal's consent policy.

## Data Availability

The data that support the findings of this study are available on request from the corresponding author. The data are not publicly available due to privacy or ethical restrictions.
